# The predictive value of diabetic retinopathy on subsequent diabetic nephropathy in patients with type 2 diabetes: a systematic review and meta-analysis of prospective studies

**DOI:** 10.1080/0886022X.2020.1866010

**Published:** 2021-01-21

**Authors:** Yu Li, Xiaoxuan Su, Qing Ye, Xiaodan Guo, Bo Xu, Tianjun Guan, Anqun Chen

**Affiliations:** a Division of Nephrology, Zhongshan Hospital, Xiamen University, Xiamen, PR China; b Teaching Hospital of Fujian Medical University, Xiamen, PR China

**Keywords:** Diabetic retinopathy, diabetic nephropathy, type 2 diabetes, meta-analysis

## Abstract

This systematic review and meta-analysis aimed to assess the predictive value of diabetic retinopathy (DR) on further diabetic nephropathy (DN) risk in patients with type 2 diabetes (T2D) based on the prospective cohort studies. PubMed, Embase, and the Cochrane Library were systematically searched for eligible prospective cohort studies through March 2020. The predictive value of DR was assessed using sensitivity, specificity, positive likelihood ratio (PLR) and negative likelihood ratio (NLR), diagnostic odds ratio (DOR), and area under the receiver operating characteristic curve (AUC) through the bivariate generalized linear mixed model and the random-effects model. Ten prospective cohort studies recruited 635 patients with T2D. The pooled sensitivity and specificity of DR for predicted DN were noted to be 0.64 (95% CI, 0.54–0.73) and 0.77 (95% CI, 0.60–0.88), respectively. The pooled PLR and NLR of DR for predicted DN were 2.72 (95% CI, 1.42–5.19) and 0.47 (95% CI, 0.33–0.67), respectively. The summary DOR for the relationship between DR and subsequent DN for T2D patients was 5.53 (95% CI, 2.00–15.30), and the AUC of DR for predicted DN was 0.73 (95% CI, 0.69–0.77). This study found significant associations between DR and subsequent DN risk for patients with T2D. Moreover, the predictive value of DR on subsequent DN risk was relatively lower.

## Introduction

Diabetes is one of the most crucial global health problems. Nearly 425 million diabetic patients exist across the world and this figure is expected to rise to 693 million by 2045 [1,2]. Patients with diabetes are characterized by hyperglycemia, metabolic abnormalities, and complications on the blood vessels, eyes, kidneys, and nerves [3]. More than 80% of diabetic patients have type 2 diabetes (T2D), which is characterized by slow onset, heterogeneous disorder, and effects of environmental factors and polygenetic inheritance [4]. Diabetic nephropathy (DN), as a common complication for diabetic patients, is increasing owing to the rapid rise of the prevalence of T2D, which accounts as the leading cause of chronic kidney and end-stage renal diseases [5]. A study has already demonstrated that hyperglycemia was an independent risk factor for the progression of diabetic retinopathy (DR) and DN, and patients presenting with each complication could progress to other complications [6].

The progression of DR and DN for patients with T2D was discordant. The Renal Insufficiency and Cardiovascular Events study found that the progression of DN did not affect 41.4% of T2D patients with advanced DR [7]. Glycemic variability over the long term was found not to affect the progression of DR but could predict the presence of DN [8]. These results could be explained by the pathogenesis of DR and DN which could be affected by different risk factors. Moreover, the previous meta-analysis has already found that DR should be regarded as a useful status for diagnosing and predicting DN for patients with T2D. However, this result could mostly bias the available evidence designed as retrospective studies [9]. Therefore, the current systematic review and meta-analysis were conducted to assess the predictive value of DR on the progression of DN for patients with T2D based on prospective cohort studies.

## Materials and methods

### Data sources, search strategy, and selection criteria

The preferred reporting items for systematic reviews and meta-analysis statement issued in 2009 was applied to guide the performance and report of this study [10]. Study designed as prospective cohort and investigated the role of DR on the progression of DN for patients with T2D were eligible in our meta-analysis. No restrictions were placed on publication language and status. PubMed, Embase, and the Cochrane Library were systematically searched through March 2020 for the use of the terms (biopsy or pathology) AND DN AND DR AND (diagnosis or etiology or pathology). The details regarding the search strategies in PubMed, Embase, and the Cochrane Library are summarized in Supplemental 1. The reference lists from relevant review and studies were also manually searched to identify any new study meeting the inclusion criteria. Moreover, relevant articles that cited retrieved studies were reviewed to check if the data was not available in original articles.

The literature search and study selection were performed by two reviewers (LY and SX). Disagreements between reviewers were resolved by a discussion until a consensus is reached. The inclusion criteria in this study are (1) patients (all of the patients diagnosed with T2D), (2) exposure and control (DR and non-DR patients with T2D), (3) gold reference (DN based on kidney biopsy findings) (4) outcomes (true/false positive and true/false negative), and (5) study design (all study designed prospective cohort). Studies with retrospective designs were excluded to avoid uncontrolled selection and confounder biases.

### Data collection and quality assessment

Two reviewers independently performed data abstraction and quality assessment (YQ and GX). Moreover, inconsistencies between reviewers were settled by an additional reviewer (CA) who conducted full-text evaluations. The following items were abstracted: first authors’ surname, publication year, country, sample size, mean age, male percentage, duration of diabetes, DR diagnostic criteria, DN diagnostic criteria, true/false positive, and true/false negative. The Newcastle–Ottawa Scale (NOS) was applied to assess the quality of included studies based on selection (four items), comparability (one item), and outcome (three items) [11]. The staring system of NOS for each study ranged from 0 to 9. Studies with seven or more stars were considered as high quality.

### Statistical analysis

The sensitivity, specificity, positive likelihood ratio (PLR), negative likelihood ratio (NLR), diagnostic odds ratio (DOR), and area under the receiver operating characteristic curve (AUC) were calculated based on the true/false positive and true/false negative in each study before data pooling. To assess the pooled predictive parameters, the bivariate generalized linear mixed model and random-effects model were then applied [12–14]. Heterogeneity across included studies was assessed using the *I*^2^ and Q statistics. Significant heterogeneity was defined as *p* < .10 [15]. Sensitivity analysis was also conducted to assess the stability of DOR for the role of DR on the risk of DN in patients with T2D [16]. Subgroup analyses for sensitivity, specificity, PLR, NLR, DOR, and AUC were also conducted based on the country, mean age, male percentage, and study quality. On the other hand, the differences between subgroups were also assessed using the interaction *P* test [17]. Publication bias was also assessed by using the funnel plot and Deeks’ asymmetry test [18]. The inspection level was two-sided, and *p* < .05 was regarded as statistically significant. The STATA software version 10.0 (Stata Corporation, College Station, TX) was applied in the conduct of all statistical analyses.

## Results

### Literature search

**Figure 1.**
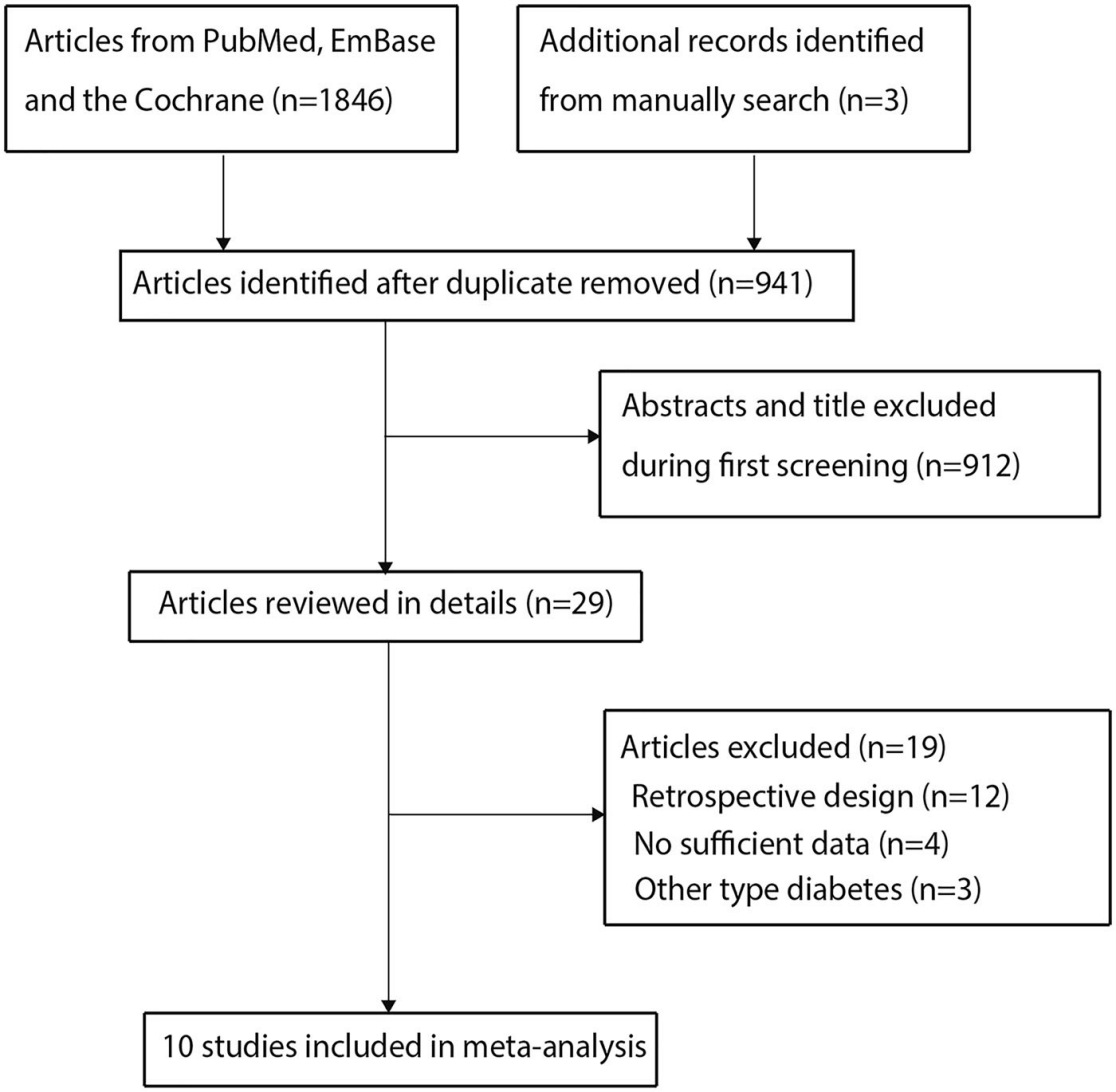
Flowchart of literature search and the selection process of the studies.

A total of 1846 articles were identified through systematically searching the predefined electronic databases and 941 articles were retained after duplicate studies were excluded. However, 912 studies were further excluded owing to the reporting of irrelevant topics. The remaining 29 studies were retrieved for further full-text evaluations. Furthermore, 19 studies were excluded owing to the retrospective design (*n* = 12), insufficient data (*n* = 4), and other types of diabetes (*n* = 3). Reviewing the reference lists of the remaining 10 studies did not yield a new study that met the inclusion criteria. Finally, 10 prospective cohort studies were selected for the final meta-analysis (Figure 1) [19–28].

### Study characteristics

The baseline characteristics of the included studies and enrolled patients are shown in Table 1. These studies were published between 1994 and 2015, and 34–110 patients were included in each individual study. The mean age of included patients ranged from 46.3 to 59 years, and the male percentage ranged from 41.3% to 94.1%. Four and six studies were conducted in Europe and Asia, respectively. Study quality was assessed by using the NOS; two studies had eight stars, four studies had seven stars, and the remaining four studies had six stars.

**Table 1.** Baseline characteristic of included studies.

Study	Country	Sample size	Mean age (years)	Percentage male (%)	Duration of diabetes	DR diagnostic criteria	DN diagnostic criteria	True positive	False positive	False negative	True negative	NOS score
[19]	India	80	47.4	60.0	Not mentioned	Fundoscopy by a qualified ophthalmologist using a clinical ophthalmoscope	Diffuse intercapillary glomerulosclerosis	6	7	8	53	7
[20]	Italy	53	NA	66.0	7–14 years	Ophthalmoscopy after mydriasis	‘typical’ diabetic glomerulopathy by light microscopy	14	15	0	24	8
[21]	China	51	54.7	70.6	7.2 years	Fundoscopy by the attending physician or ophthalmologist with pupils adequately dilated: presence of background retinopathy with or without proliferative changes	The presence of mesangial expansion and diffuse intercapillary glomerulosclerosis	20	10	14	7	6
[22]	Denmark	34	52.5	94.1	13–18 years	Direct ophthalmoscopy	Biopsy-proven diabetic glomerulosclerosis	17	0	9	8	6
[23]	Japan	109	48.1	67.9	7.3 years	Direct ophthalmoscopy with pupils dilated by an ophthalmologist	Pathological evaluation of renal biopsy specimens, including light microscopy, electron microscopy, and immunofluorescence staining	46	8	24	20	7
[24]	Denmark	49	54.7	81.6	5.0 years	Direct ophthalmoscopy after pupillary dilation and afterwards by fundus photography and graded: nil, simplex, or proliferative retinopathy	Diffuse diabetic glomerulosclerosis, hyaline arteriolosclerosis, tubular atrophy, and interstitial fibrosis	17	11	17	4	6
[25]	China	68	48.7	55.9	6.4 years	Presence of proliferative or background changes. About 60–70% of patients were assessed by an ophthalmologist and the rest by physician alone.	Presented 3 of following features: morphological criteria of diabetic glomerular lesions included glomerular Hyaline arteriolosclerosis; global mesangial sclerosis with or without Kimmelstiel–Wilson nodule or nodular mesangial sclerosis; exudative lesions such as ‘fibrin cap’, ‘capsular drop’, or ‘hyaline thrombus’; microaneurysm; uniform glomerular capillary basement membrane thickening; or ultrastructural thickness 350 nm	17	8	7	36	7
[26]	Spain	35	59.0	62.9	Not mentioned	Formal ophthalmological examination and fluorescein angiography was performed in all the patients.	Glomerulosclerosis either of the nodular or diffuse types, interstitial fibrosis with infiltration by mononuclear inflammatory cells, hyalinization of the renal arterioles, linear deposits of Igs (Immunoglobulins) in glomerular basement membrane on immunofluorescence studies and diffuse thickening of glomerular basement membrane on electron microscopy examination	9	3	17	6	7
[27]	China	110	46.3	69.7	5.0 years	Not mentioned	Diffuse mesangial expansion with predominance of increased mesangial matrix, Kimmelstiel–Wilson nodular lesions, hyaline exudative lesions, and glomerular basement membrane thickening	46	5	14	45	8
[28]	Iran	46	48.9	41.3	7.2 years	Background ± proliferative changes on funduscopy and/or fluorescein angiography	Kidney biopsy samples were examined by light microscopy, immunofluorescence, and electron microscopy	17	1	9	19	6

### Sensitivity and specificity

**Figure 2.**
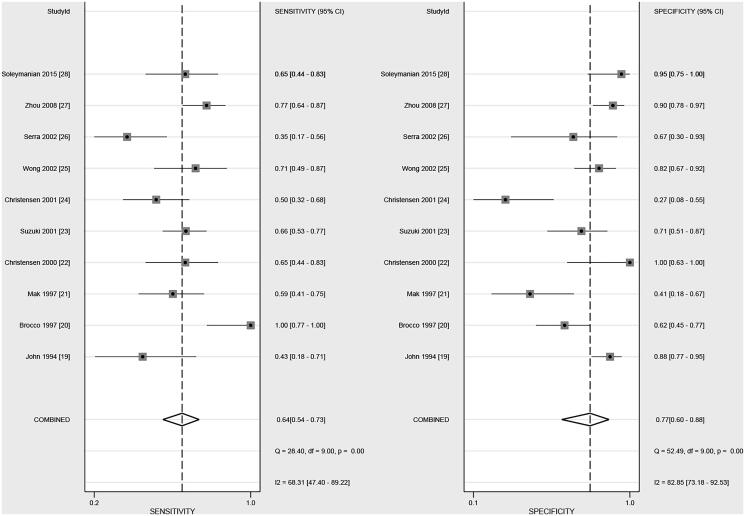
The summary sensitivity and specificity of DR on subsequent DN in patients with T2D.

The pooled sensitivity and specificity were noted to be 0.64 (95% CI, 0.54–0.73) and 0.77 (95% CI, 0.60–0.88), respectively, after pooling all of the included studies (Figure 2). Significant heterogeneity (*I*^2^=68.3%; *p* < .01) and specificity (*I*^2^=82.9%; *p* < .01) were noticed across included studies. Subgroup analysis suggested that the sensitivity of DR in younger patients was higher than in elderly patients (*p* = .032; Table 2). However, country, mean age, male percentage, and study quality did not affect the specificity of DR for predicted DN in patients with T2D (Table 2).

**Table 2.** Subgroup analyses.

Parameters	Factors	Subgroup	Effect estimate and 95%CI	*I*^2^ (%)	*p* Value for Q statistic	Ratio between subgroups	*p* Value between subgroups
Sensitivity	Country	Europe	0.67 (0.32–0.90)	83.4	<.01	1.00 (0.59–1.69)	1.000
		Asia	0.67 (0.60–0.74)	29.5	.21		
	Mean age (years)	≥50.0	0.53 (0.42–0.63)	47.5	.13	0.78 (0.62–0.98)	.032
		<50.0	0.68 (0.61–0.75)	38.3	.17		
	Percentage male (%)	≥65.0	0.67 (0.58–0.75)	66.7	.01	1.24 (0.90–1.72)	.193
		<65.0	0.54 (0.38–0.69)	65.7	.03		
	Study quality	High	0.69 (0.45–0.85)	80.1	<.01	1.15 (0.80–1.66)	.452
		Low	0.60 (0.49–0.70)	0.0	.51		
Specificity	Country	Europe	0.65 (0.32–0.88)	76.0	.01	0.79 (0.47–1.34)	.389
		Asia	0.82 (0.67–0.91)	80.2	<.01		
	Mean age (years)	≥50.0	0.62 (0.24–0.89)	76.6	.01	0.72 (0.37–1.39)	0.331
		<50.0	0.86 (0.79–0.91)	48.0	.10		
	Percentage male (%)	≥65.0	0.67 (0.43–0.85)	84.7	<.01	0.78 (0.55–1.10)	.161
		<65.0	0.86 (0.78–0.91)	38.1	.18		
	Study quality	High	0.81 (0.71–0.88)	68.5	.01	1.04 (0.53–2.02)	.912
		Low	0.78 (0.26–0.97)	88.4	<.01		
PLR	Country	Europe	1.93 (0.67–5.56)	72.0	<.01	0.52 (0.15–1.83)	.308
		Asia	3.72 (1.87-7.38)	71.1	<.01		
	Mean age (years)	≥50.0	1.38 (0.46–4.12)	15.5	.06	0.29 (0.09–0.94)	.038
		<50.0	4.79 (3.12–7.36)	4.2	.08		
	Percentage male (%)	≥65.0	2.02 (0.95–4.28)	79.8	<.01	0.53 (0.21–1.35)	.185
		<65.0	3.78 (2.20–6.50)	11.9	.07		
	Study quality	High	3.54 (2.35–5.32)	12.5	.05	1.32 (0.19–9.03)	.780
		Low	2.69 (0.41–17.70)	79.6	<.01		
NLR	Country	Europe	0.50 (0.16–1.58)	79.7	<.01	1.25 (0.38–4.07)	.711
		Asia	0.40 (0.30–0.53)	63.8	.02		
	Mean age (years)	≥50.0	0.77 (0.37–1.60)	77.2	<.01	2.08 (0.96–4.50)	.062
		<50.0	0.37 (0.29–0.47)	57.1	.05		
	Percentage male (%)	≥65.0	0.50 (0.29–0.84)	78.6	<.01	0.94 (0.49–1.80)	0.860
		<65.0	0.53 (0.37–0.77)	66.8	.03		
	Study quality	High	0.39 (0.20–0.73)	73.5	<.01	0.75 (0.29–1.92)	.548
		Low	0.52 (0.26–1.01)	79.3	<.01		
DOR	Country	Europe	3.57 (0.35–36.20)	81.0	<.01	0.46 (0.04–5.85)	.553
		Asia	7.68 (2.78–21.24)	73.2	<.01		
	Mean age (years)	≥50.0	1.19 (0.32–4.38)	62.0	.05	0.11 (0.02–0.50)	.004
		<50.0	11.06 (4.97–24.59)	50.8	.09		
	Percentage male (%)	≥65.0	5.20 (1.09–24.77)	85.9	<.01	0.81 (0.11–5.94)	.835
		<65.0	6.43 (1.86–22.22)	63.1	.04		
	Study quality	High	7.81 (3.06–19.95)	65.9	.01	2.26 (0.22–23.19)	.493
		Low	3.46 (0.41–29.20)	83.6	<.01		
AUC	Country	Europe	0.71 (0.67–0.75)	–	–	0.97 (0.90–1.05)	.492
		Asia	0.73 (0.68–0.76)	–	–		
	Mean age (years)	≥50.0	0.55 (0.50–0.59)	–	–	0.71 (0.64–0.77)	<.001
		<50.0	0.78 (0.74–0.81)	–	–		
	Percentage male (%)	≥65.0	0.70 (0.66–0.74)	–	–	0.81 (0.76–0.87)	<.001
		<65.0	0.86 (0.82–0.88)	–	–		
	Study quality	High	0.83 (0.79–0.86)	–	–	1.32 (1.22–1.42)	<.001
		Low	0.63 (0.59–0.67)	–	–		

### Positive and negative likelihood ratio

**Figure 3.**
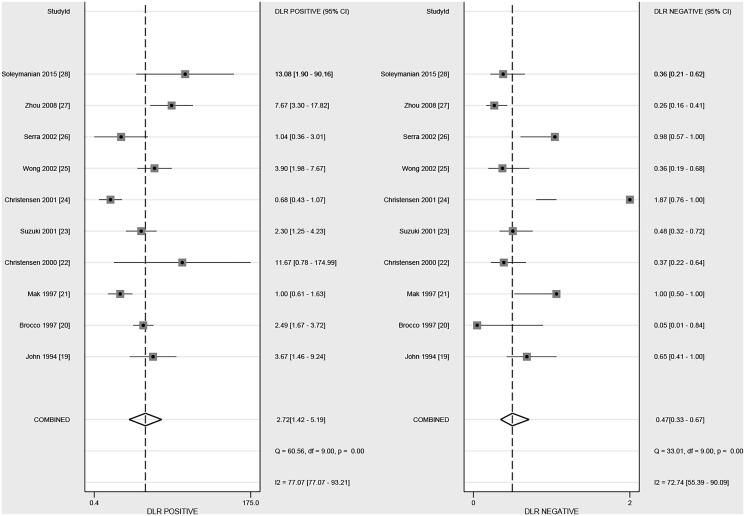
The summary PLR and NLR of DR on subsequent DN in patients with T2D.

After pooling all of the included studies, the summary PLR and NLR of DR for predicted DN in patients with T2D were 2.72 (95% CI, 1.42–5.19) and 0.47 (95% CI, 0.33–0.67), respectively (Figure 3). Moreover, significant heterogeneity among included studies for PLR (*I*^2^=77.1%; *p* < .01) and NLR (*I*^2^=72.7%; *p* < .01) were detected. Subgroup analysis suggested that the PLR in younger patients was higher than in elderly patients (*p* = .038; Table 2). However, country, mean age, male percentage, and study quality did not affect the NLR of DR for predicted DN in patients with T2D (Table 2).

### Diagnostic odds ratio

**Figure 4.**
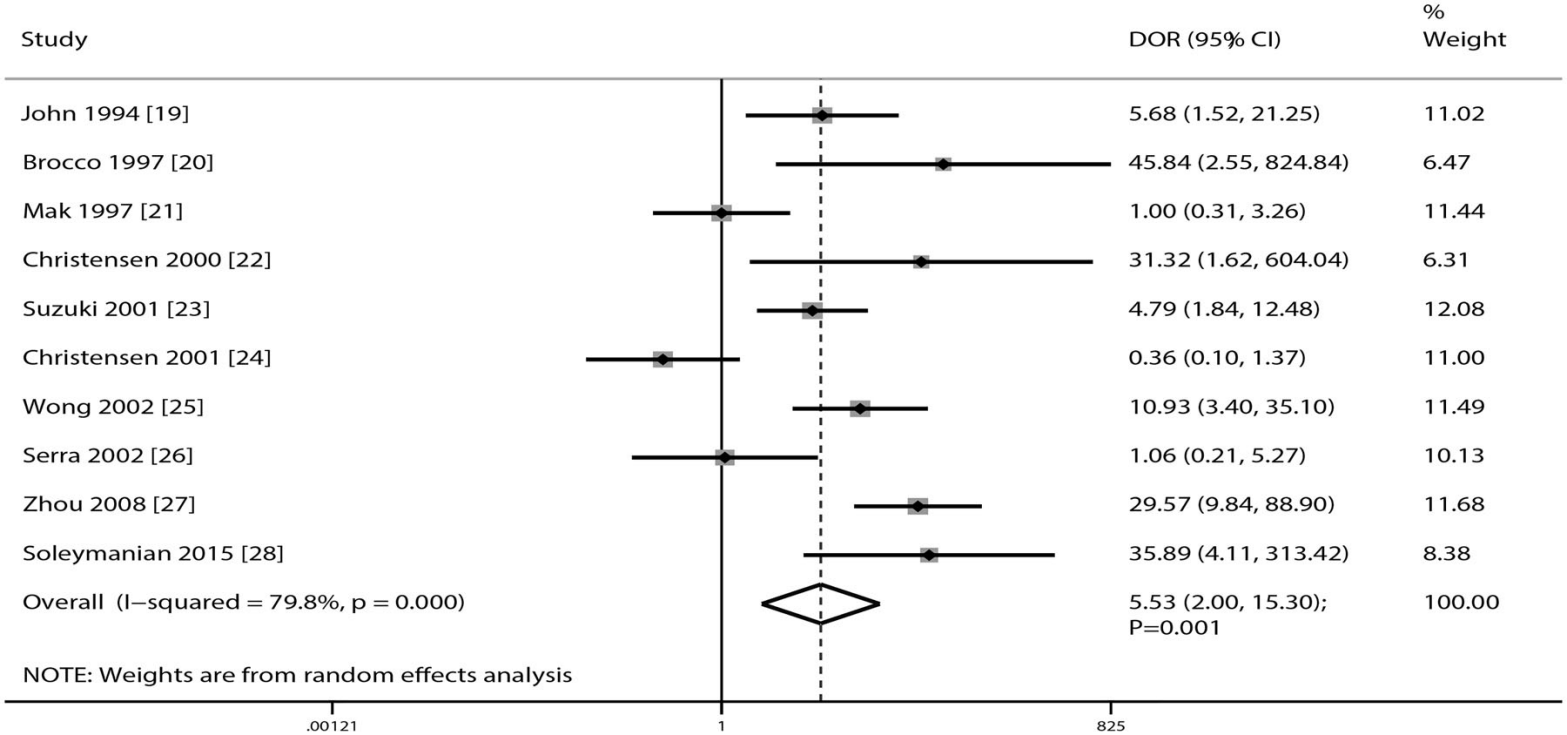
The summary DOR of DR on subsequent DN in patients with T2D.

After pooling all of the included studies, DR was noted to be associated with an increased risk of DN in patients with T2D (DOR, 5.53; 95% CI, 2.00–15.30; *p* = .001; Figure 4). Moreover, significant heterogeneity exists across included studies (*I*^2^=79.8%; *p* < .01). Sensitivity analysis suggested the pooled conclusion of robustness that was not altered by the sequential exclusion of individual study (data not shown). Subgroup analysis suggested that the DOR in younger patients was significantly higher than in elderly patients (*p* = .004; Table 2).

### Area under the receiver operating characteristic curve

**Figure 5.**
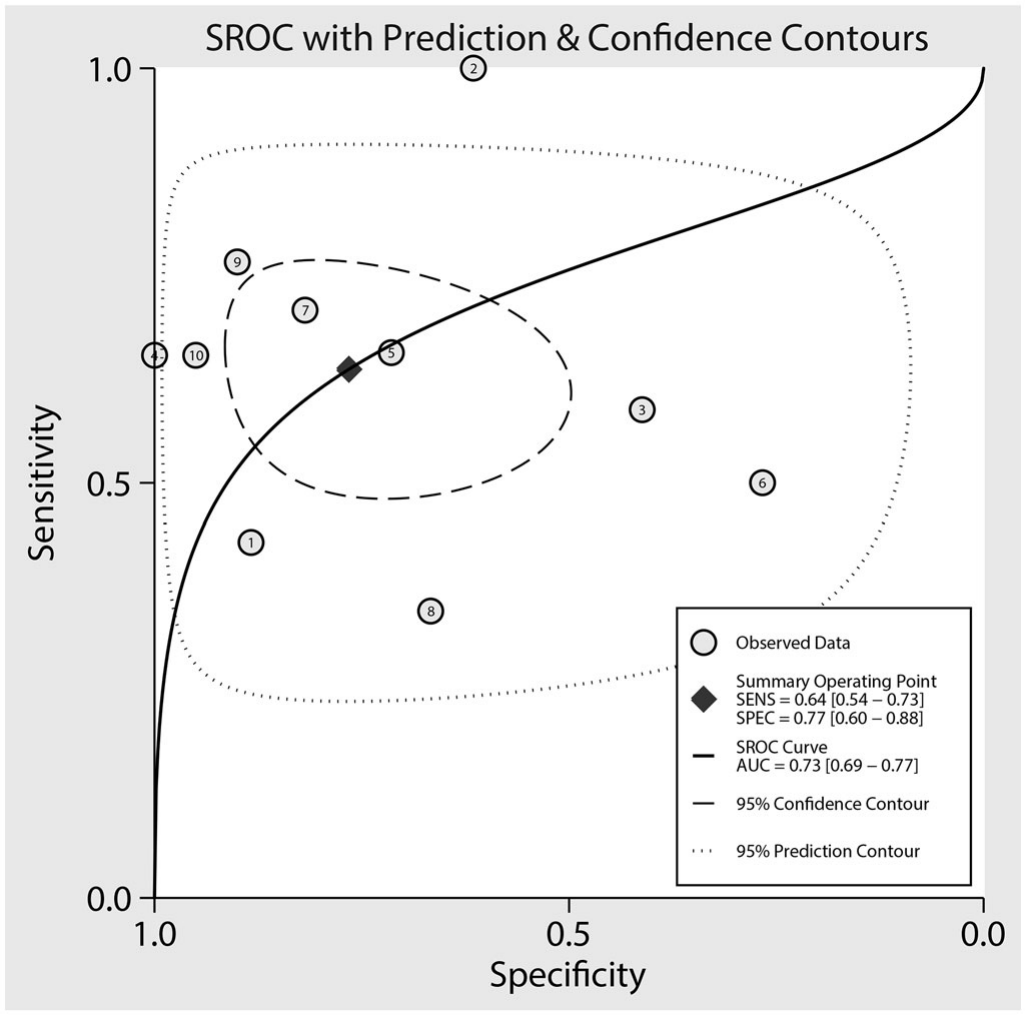
The summary AUC on subsequent DN in patients with T2D.

The summary AUC of DR for predicted DN in patients with T2D was 0.73 (95% CI, 0.69–0.77; Figure 5). Subgroup analyses found that the AUC for predicting DN was high in pooled studies with younger mean age (*p* < .001), male percentage <65.0% (*p* < .001), or study with high quality (*p* < .001; Table 2).

### Publication bias

**Figure 6.**
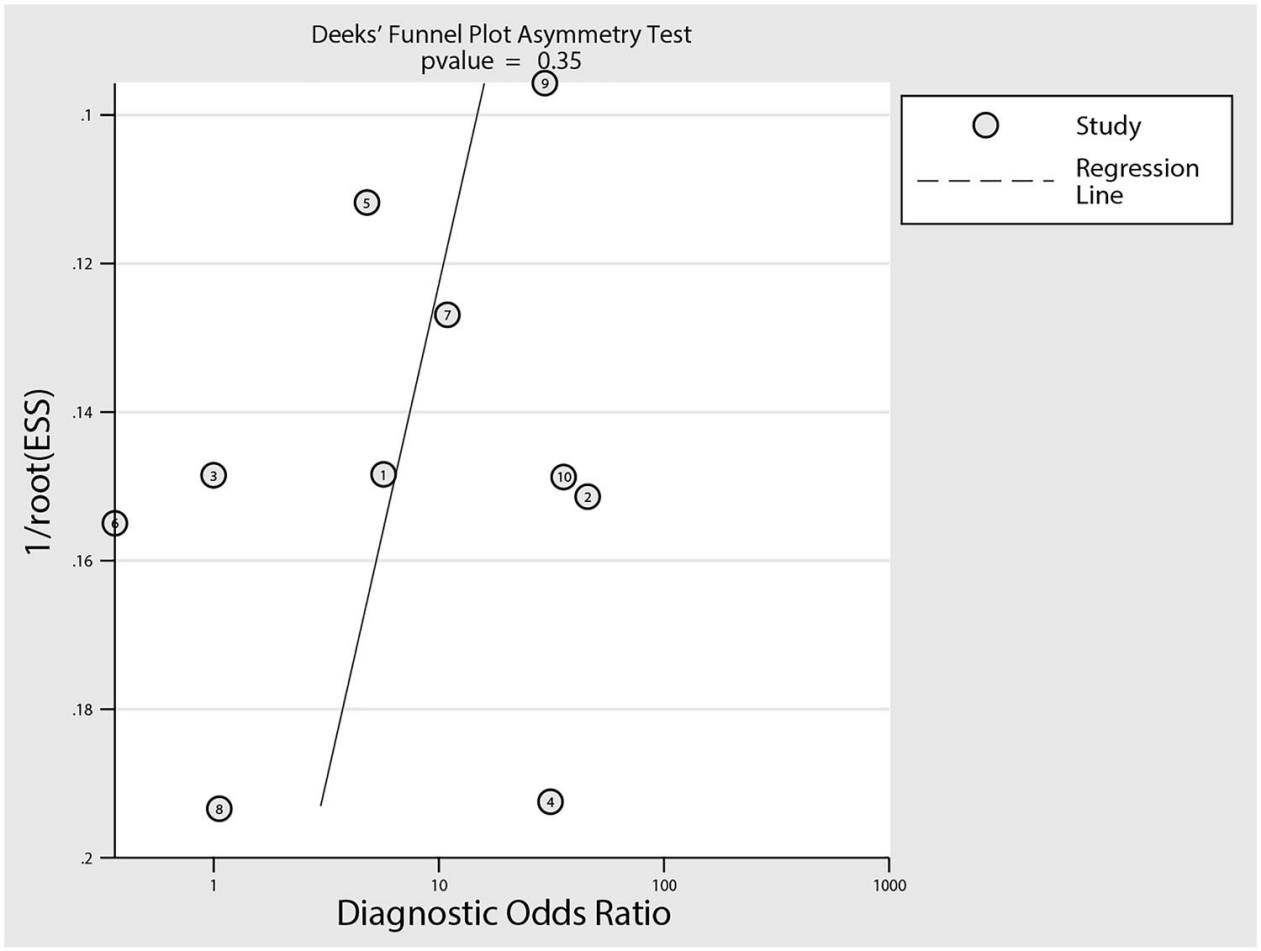
Publication bias.

Reviewing the funnel plot could not rule out the potential publication bias of DR for predicted DN in patients with T2D (Figure 6). Moreover, the Deeks’ test detected no significant publication bias (*p* = .35).

## Discussion

The complications of T2D contribute to a series of global health problems and will remain so because patients have so far been relatively easily attacked by long-term T2D duration. With the use of published data from 10 prospective cohort studies, this systematic review and meta-analysis have identified the predictive value of DR on subsequent DN for patients with T2D. This study included 635 patients with T2D across a wide range of characteristics. In addition, this study found that the predictive value of DR on subsequent DN for patients with T2D was mild. Moreover, the predictive value of DR could be affected by mean age, male percentage, and study quality. Finally, the mean age of patients should be introduced as an important factor because it could affect sensitivity, PLR, DOR, and AUC. However, the male percentage and study quality affected just the AUC of DR for predicted DN in patients with T2D.

A previous meta-analysis included 26 studies and found that the sensitivity and specificity of DR for predicted DN were 0.65 and 0.75, respectively. Moreover, the PLR and NLR of NR to predict DN were 0.72 and 0.69, respectively. The DOR and AUC were 5.67 and 0.75, respectively [9]. However, this study combined both prospective and retrospective studies, and the selection and confounder biases could affect the predictive value of DR on the progression of DN in T2D. Moreover, a significant heterogeneity was seen across the included studies. However, sensitivity and subgroup analyses were not conducted to explore the source of heterogeneity. Therefore, the current systematic review and meta-analysis were conducted to assess the predictive value of DR on the progression of DN in patients with T2D. Moreover, the predictive value of DR in patients with specific characteristics was also assessed.

The summary result of this study found a significant association between DR and subsequent DN in patients with T2D. Although most included studies reported similar conclusions, three of the included studies reported inconsistent results [21,24,26]. A study conducted by Mak et al. found that microscopic hematuria and non-nephrotic proteinuria could predict the progression of nondiabetic renal disease for patients with T2D while DR was not associated with the risk of DN in T2D patients [21]. This result could be explained by a similar duration of T2D and the degree of diabetes control. Christensen et al. found no significant association between DR and DN in patients with T2D [24]. This result may correlate with potential selection bias because the characteristics of the patients between groups were not balanced. Serra et al. conducted a prospective cohort study of 35 patients with T2D and found that DN contributed to an important renal lesion in T2D patients with biopsied proteinuria, irrespective of the characteristics of microhematuria and retinopathy, which may be correlated with most patients having diabetic glomerulopathy or normal glomeruli regarded as DN [26] .

The predictive value of DR on subsequent DN for patients with T2D was mild, and the pooled sensitivity and specificity were 0.64 and 0.77, respectively. Moreover, the PLR and NLR of DR on DN in T2D patients were 2.72 and 0.47, respectively. Moreover, significant heterogeneity was seen across included studies, which could be explained by various baseline characteristics and ethnic origin [29]. Subgroup analysis found that the predictive value of DR on subsequent DN could be affected by mean age, male percentage, and study quality. Moreover, the mean age of patients was an important independent factor, which could bias the predictive value of DR on DN in patients with T2D. The potential reason for this could be that the age of the patients significantly correlates with the severity of disease, glucose control, and prevalence of diabetic complications.

Several strengths of this study should be highlighted. First, the analysis of this study was based on prospective cohort studies, and the selection and recall biases could be minimized by retrospective studies. Second, this study was based on 10 published studies, and the stability of the pooled conclusion was superior to any individual study. Third, stratified analyses for sensitivity, specificity, PLR, NLR, DOR, and AUC were also conducted to assess whether the predictive value of DR on subsequent DN could be affected by patients’ characteristics. However, the limitations of this study should be acknowledged. First, several characteristics of included patients were not available, such as the background of genetic and antidiabetic treatment regimens, or other comorbidity diseases, which could affect the progression of DN in patients with T2D. Second, the heterogeneity across included studies for diagnostic parameters was not fully explained by using the sensitivity and subgroup analyses. Third, this study was not registered and transparency was restricted. Fourth, the selection bias potentially existed because the patients tended to undergo regular retinopathy and nephropathy follow-up in most well-designed cohort studies, whereas patients with poor compliance had been easily excluded. Fifth, the development and progression of DR and DN in T2D patients differ, and the causality relationship was not obtained. Sixth, the duration of T2D could affect the progression of DN, whereas subgroup analysis was not performed because numerous studies did not give the mean duration of T2D. Seventh, publication bias was inevitable because the analysis was based on published articles, and unpublished data were not available. Lastly, the limitation of any traditional meta-analysis, including the analysis based on pooled data, restricted the conduct of more detailed analyses.

In conclusion, this study found that DR was associated with an increased risk of DN in patients with T2D, and the predictive value of DR on subsequent DN risk for T2D patients was relatively lower. Moreover, the predictive value of DR could be affected by mean age, male percentage, and study quality. The mean age of the patients could be affected by sensitivity, PLR, DOR, and AUC. The predictive value of DR on other diabetic complications should be evaluated in further large-scale prospective cohort studies.

## Supplementary Material

Supplemental Material

## Data Availability

The authors confirm that the data supporting the findings of this study are available within the article.

## Geolocation information

No. 201-209 Hubin South Road, Siming District, Xiamen, Fujian, China.

## References

[1] Zimmet PZ, Magliano DJ, Herman WH, et al. Diabetes: a 21st century challenge. Lancet Diabetes Endocrinol. 2014;2(1):56–64. 24622669 10.1016/S2213-8587(13)70112-8

[2] Cho NH, Shaw JE, Karuranga S, et al. IDF diabetes atlas: global estimates of diabetes prevalence for 2017 and projections for 2045. Diabetes Res Clin Pract. 2018;138:271–281. 29496507 10.1016/j.diabres.2018.02.023

[3] Powers AC, Niswender KD, Rickels MR, et al. Diabetes mellitus. In: Longo DL, Fauci AS, Kasper DL, editors. Harrison’s principles of internal medicine. 18th ed. New York (NY): McGraw-Hill; 2012. p. 2968–3002.

[4] Ostenson CG. The pathophysiology of type 2 diabetes mellitus: an overview. Acta Physiol Scand. 2001;171(3):241–247. 11412136 10.1046/j.1365-201x.2001.00826.x

[5] Ritz E, Rychlík I, Locatelli F, et al. End-stage renal failure in type 2 diabetes: a medical catastrophe of worldwide dimensions. Am J Kidney Dis. 1999;34(5):795–808. 10561134 10.1016/S0272-6386(99)70035-1

[6] Kramer CK, Retnakaran R. Concordance of retinopathy and nephropathy over time in Type 1 diabetes: an analysis of data from the Diabetes Control and Complications Trial. Diabet Med. 2013;30(11):1333–1341. 23909911 10.1111/dme.12296

[7] Penno G, Solini A, Zoppini G, et al. Rate and determinants of association between advanced retinopathy and chronic kidney disease in patients with type 2 diabetes: the Renal Insufficiency and Cardiovascular Events (RIACE) Italian multicenter study. Diabetes Care. 2012;35(11):2317–2323. 23093684 10.2337/dc12-0628 PMC3476898

[8] Penno G, Solini A, Bonora E, et al. HbA1c variability as an independent correlate of nephropathy, but not retinopathy, in patients with type 2 diabetes: the renal insufficiency and cardiovascular events (RIACE) Italian multicenter study. Diabetes Care. 2013;36(8):2301–2310. 23491522 10.2337/dc12-2264 PMC3714498

[9] He F, Xia X, Wu XF, et al. Diabetic retinopathy in predicting diabetic nephropathy in patients with type 2 diabetes and renal disease: a meta-analysis. Diabetologia. 2013;56(3):457–466. 23232641 10.1007/s00125-012-2796-6

[10] Moher D, Liberati A, Tetzlaff J, PRISMA Group, et al. Preferred reporting items for systematic reviews and meta-analyses: the PRISMA statement. PLoS Med. 2009;6(7):e1000097. 19621072 10.1371/journal.pmed.1000097 PMC2707599

[11] Wells G, Shea B, O’Connell D, et al. The Newcastle–Ottawa Scale (NOS) for assessing the quality of non-randomized studies in meta-analysis. (cited 2020 May 01, 2020) Available from: http://www.ohri.ca/programs/clinical_epidemiology/oxford.asp.

[12] DerSimonian R, Laird N. Meta-analysis in clinical trials. Control Clin Trials. 1986;7(3):177–188. 3802833 10.1016/0197-2456(86)90046-2

[13] Walter SD. Properties of the summary receiver operating characteristic (SROC) curve for diagnostic test data. Stat Med. 2002;21(9):1237–1256. 12111876 10.1002/sim.1099

[14] Ades AE, Lu G, Higgins JP. The interpretation of random-effects meta-analysis in decision models. Med Decis Making. 2005;25(6):646–654. 16282215 10.1177/0272989X05282643

[15] Deeks JJ, Higgins JPT, Altman DG. Chapter 10: analysing data and undertaking meta-analyses. In: Higgins JPT, Thomas J, Chandler J, et al, editors. Cochrane handbook for systematic reviews of interventions. 2nd ed. Chichester (UK): John Wiley & Sons; 2019. p. 241–284.

[16] Tobias A. Assessing the influence of a single study in the meta-analysis estimate. Stata Tech Bull. 1999;8(47):15–17.

[17] Woodward M. Epidemiology: study design and data analysis. 3rd ed. Boca Raton (FL): CRC Press; 2013.

[18] Deeks JJ, Macaskill P, Irwig L. The performance of tests of publication bias and other sample size effects in systematic reviews of diagnostic test accuracy was assessed. J Clin Epidemiol. 2005;58(9):882–893. 16085191 10.1016/j.jclinepi.2005.01.016

[19] John GT, Date A, Korula A, et al. Nondiabetic renal disease in noninsulin-dependent diabetics in a south Indian Hospital. Nephron. 1994;67(4):441–443. 7969678 10.1159/000188019

[20] Brocco E, Fioretto P, Mauer M, et al. Renal structure and function in non-insulin dependent diabetic patients with microalbuminuria. Kidney Int Suppl. 1997;63:S40–S44. 9407419

[21] Mak SK, Gwi E, Chan KW, et al. Clinical predictors of non-diabetic renal disease in patients with non-insulin dependent diabetes mellitus. Nephrol Dial Transplant. 1997;12(12):2588–2591. 9430856 10.1093/ndt/12.12.2588

[22] Christensen PK, Gall MA, Parving HH. Course of glomerular filtration rate in albuminuric type 2 diabetic patients with or without diabetic glomerulopathy. Diabetes Care. 2000;23(2):B14–20. 10860186

[23] Suzuki D, Takano H, Toyoda M, et al. Evaluation of renal biopsy samples of patients with diabetic nephropathy. Intern Med. 2001;40(11):1077–1084. 11757760 10.2169/internalmedicine.40.1077

[24] Christensen PK, Larsen S, Horn T, et al. Renal function and structure in albuminuric type 2 diabetic patients without retinopathy. Nephrol Dial Transplant. 2001;16(12):2337–2347. 11733625 10.1093/ndt/16.12.2337

[25] Wong TY, Choi PC, Szeto CC, et al. Renal outcome in type 2 diabetic patients with or without coexisting nondiabetic nephropathies. Diabetes Care. 2002;25(5):900–905. 11978688 10.2337/diacare.25.5.900

[26] Serra A, Romero R, Bayés B, et al. Is there a need for changes in renal biopsy criteria in proteinuria in type 2 diabetes? Diabetes Res Clin Pract. 2002;58(2):149–153. 12213357 10.1016/s0168-8227(02)00131-6

[27] Zhou J, Chen X, Xie Y, et al. A differential diagnostic model of diabetic nephropathy and non-diabetic renal diseases. Nephrol Dial Transplant. 2008;23(6):1940–1945. 18156459 10.1093/ndt/gfm897

[28] Soleymanian T, Hamid G, Arefi M, et al. Non-diabetic renal disease with or without diabetic nephropathy in type 2 diabetes: clinical predictors and outcome. Ren Fail. 2015;37(4):572–575. 25682971 10.3109/0886022X.2015.1007804

[29] Stolk RP, van Schooneveld MJ, Cruickshank JK, et al. Retinal vascular lesions in patients of Caucasian and Asian origin with type 2 diabetes: baseline results from the ADVANCE retinal measurements (AdRem) study. Diabetes Care. 2008;31(4):708–713. 18184903 10.2337/dc07-1657

