# Supplementary material for: The predictive value of diabetic retinopathy on subsequent diabetic nephropathy in patients with type 2 diabetes: a systematic review and meta-analysis of prospective studies
Source: Ren Fail. 2021 Jan 21;43(1):231–40. doi: 10.1080/0886022X.2020.1866010 (PMC7833016; doi:10.1080/0886022X.2020.1866010)
Supplement: Supplemental Material [file IRNF_A_1866010_SM0289.docx]

1. **Search strategy in PubMed and Cochrane library:**

# #1. (“Diabetes mellitus, type 2” [Mesh] or “type 2 diabetes” [Title/Abstract] or “type ii diabetes” [Title/Abstract])

# #2. (biopsy [Title/Abstract] or pathology [Title/Abstract])

# #3. (“Diabetic Nephropathies” [Mesh] or “diabetic nephropathy” [Title/Abstract])

# #4. (“diabetic retinopathy” [Mesh] or “diabetic retinopathy” [Title/Abstract])

# #5. (diagnosis [Title/Abstract] or aetiology [Title/Abstract] or pathology [Title/Abstract])

# #6. #1-#5/AND NOT animals [mh].

1. **EMBASE:**

#1. (Diabetes mellitus or type 2 diabetes or type ii diabetes).af

#2. (biopsy or pathology).af

#3. (diabetic nephropathy).af

#4. (diabetic retinopathy).af

#5. (diagnosis or aetiology or pathology).af

#6. #1-#5/AND
